# Mechanism underlying painful radiculopathy in patients with lumbar disc herniation

**DOI:** 10.1002/ejp.1947

**Published:** 2022-04-12

**Authors:** Gil Samuelly‐Leichtag, Elon Eisenberg, Yaniv Zohar, Maisa Andraous, Ayelet Eran, Gill E. Sviri, Ory Keynan

**Affiliations:** ^1^ B. Rappaport Faculty of Medicine Technion, Israel Institute of Technology Haifa Israel; ^2^ Institute for Pain Medicine Rambam Health Care Campus Haifa Israel; ^3^ Pathology Laboratory Rambam Health Care Campus Haifa Israel; ^4^ Medical Imaging Division Rambam Health Care Campus Haifa Israel; ^5^ Department of Neurosurgery Rambam Health Care Campus Haifa Israel; ^6^ Division of Spine Surgery Department of Orthopedics Rambam Health Care Campus Haifa Israel

## Abstract

**Background:**

Painful lumbar radiculopathy is a neuropathic pain condition, commonly attributed to nerve root inflammation/compression by disc herniation. The present exploratory study searched for associations between pain intensity and inflammatory markers, herniated disc size, infection, psychological factors and pain modulation in patients with confirmed painful lumbar radiculopathy scheduled for spine surgery.

**Methods:**

Prior to surgery, 53 patients underwent the following evaluation: pain intensity measured on a 0–10 numeric rating scale (NRS) and the Short‐Form McGill Pain Questionnaire; sensory testing (modified DFNS protocol); pain processing including temporal summation and conditioned pain modulation (CPM); neurological examination; psychological assessment including Spielberger's Anxiety Inventory, Pain Sensitivity Questionnaire and the Pain Catastrophizing Scale. Pro‐inflammatory cytokine levels (IL‐1b, IL‐6, IL‐8, IL‐17, TNFα, IFNg) and microbial infection (ELISA and rt‐PCR) in blood and disc samples obtained during surgery. MRI scans assessments for disc herniation size/volume (MSU classification/ three‐dimensional volumetric analysis).

**Results:**

Complete data were available from 40 (75%) patients (15 female) aged 44.8 ± 16.3 years. Pain intensity (NRS) positively correlated with pain catastrophizing and CPM (*r* = 0.437, *p* = 0.006; *r* = 0.421, *p* = 0.007; respectively), but not with disc/blood cytokine levels, bacterial infection or MRI measures. CPM (*p* = 0.001) and gender (*p* = 0.029) were associated with average pain intensity (adjusted R^2^ = 0.443).

**Conclusions:**

This exploratory study suggests that pain catastrophizing, CPM and gender, seem to contribute to pain intensity in patients with painful lumbar radiculopathy. The role of mechanical compression and inflammation in determining the intensity of painful radiculopathy remains obscure.

**Significance of study:**

Pain catastrophizing, CPM and gender rather than objective measures of inflammation and imaging seem to contribute to pain in patients with painful radiculopathy.

## 1 INTRODUCTION

Painful lumbar radiculopathy is a neuropathic pain condition thought to be caused by a nerve root lesion, commonly due to disc herniation (Porchet et al., 2002; Valat et al., 2010). The underlying pain mechanisms are debatable but are often attributed to either mechanical compression of the nerve root by the herniated disc, and/or local inflammation (Erbüyün et al., 2018; Kirita et al., 2007). Indeed, animal models of mechanical nerve root compression produce mechanical allodynia, thermal hyperalgesia and hypoesthesia, similar to those found clinically in these patients. Likewise, elevated levels of pro‐inflammatory cytokines such as interleukin (IL)‐1b, IL‐6, IL‐8, IL‐17, tumour necrosis factor alpha (TNFα) and interferon gamma (IFNg) have been found in blood and disc tissue obtained during surgery in humans as well as in animal models of radiculopathy.

There is some evidence that associates painful lumbar radiculopathy with an anaerobic bacterial infection with Propionibacterium acnes, although this is more commonly seen in patients with previous back surgery (Ben‐Galim et al., 2006; Chen et al., 2016). Alterations in pain processing, quantified by dynamic psychophysical testing of temporal summation (TS) and conditioned pain modulation (CPM), have also been found in patients with low back pain. Some studies have demonstrated enhanced TS and reduced CPM in patients with low back pain (McPhee et al., 2020), while others reported mix results (den Bandt et al., 2019; Neelapala et al., 2020). Lastly, psychological factors, specifically pain catastrophizing (Haugen et al., 2012; Marshall et al., 2017) and anxiety (Fernandez et al., 2017), have been related to chronic low back pain and sciatica. All of these factors have been investigated separately to at least some degree in patients with low back pain, ‘sciatica’ or lumbar radiculopathy. However, to our knowledge, these factors have not been studied together in one well‐defined population of patients with confirmed painful radiculopathy. Furthermore, associations between each of these factors and the intensity of the neuropathic pain in such patients have not been established. Understanding of the underling mechanisms may impact clinical decision making and optimize non‐surgical treatment outcomes.

Based on a review of the literature, we hypothesized that pain intensity in patients with confirmed painful radiculopathy will be associated with more than one factor and will likely involve pathological (compression/inflammation), psychological and altered pain modulation aspects. The current exploratory study aimed to verify this hypothesis.

## 2 METHODS

### 2.1 Protocol approval and patient consents

The study (ClinicalTrials.gov Identifier: NCT03432507) was approved by the Rambam Health Care Campus Institutional Review Board (#0477‐17). Fifty‐three adult patients suffering from painful lumbar radiculopathy due to disc herniation (per CT or MRI scans) and who were candidates for lumbar spine surgery, were recruited from the Spine and Neurosurgery Clinics at Rambam between 02/18 and 07/19. Painful lumbar radiculopathy was diagnosed based on the IASP’s redefinition of neuropathic pain: a pain distribution consistent with a defined nerve root territory; negative and/or positive sensory neurological signs on examination; radiological findings confirming the presence of a herniated disc at side and level congruent with the clinical signs and symptoms (Scholz et al., 2019). Exclusion criteria were low back pain or sciatica related to causes other than a herniated disc (i.e. infection, tumour, trauma); previous lumbar spine surgery at the same level; being immunocompromised; pregnancy, and age below 18 years.

### 2.2 Study procedure

All subjects underwent MRI scans prior to their scheduled surgery. MRI scans were retrospectively assessed by a radiologist (MA) who was blinded to the patients’ clinical condition, for disc herniation size and location. After consenting to participate in the study and prior to surgery, the subjects completed the pain assessment, sensory and pain modulation testing and the psychological questionnaires. Tests were conducted at the pre‐operative clinic, typically a few days prior to surgery or upon admission to the surgical ward a day before or on the day of surgery. Patients were instructed to avoid analgesics 24 h before the assessment. In the operating room, blood samples were obtained and herniated disc tissue was collected and stored. All samples were then tested for levels of pro‐inflammatory cytokines and microbial infections.

### 2.3 Assessment of pain intensity

Patients were requested to report their pain intensity (average, minimal and maximal) during the week prior to surgery on a 0–100 numerical pain scale (NPS) (Chiarotto et al., 2019) and to complete all 15 descriptors (11 sensory; 4 affective) of the Short‐Form McGill Pain Questionnaire (SF‐MPQ) (Dworkin et al., 2015).

### 2.4 Sensory examination

Sensory examination, necessary for establishing the diagnosis of neuropathic pain, was performed by a trained physical therapist (GS) and consisted of a bedside examination and Quantitative Sensory Testing (QST). The bedside examination included the Straight Leg Raise (SLR) test performed separately in the painful and the contralateral legs and measured with a standard hand‐held goniometer, and the application of dynamic light touch and pin‐prick (safety pin) stimuli to each dermatome in the painful and contralateral legs for assessing hypoesthesia/allodynia and hypo/hyperalgesia respectively.

Quantitative Sensory Testing was based on the DFNS (German Research Network on Neuropathic Pain) protocol, (Rolke et al., 2006) although slightly modified this protocol consists of a set of nine evoked tests which measure sensory integrity and pain perception to thermal (heat and cold) and mechanical (vibration fork, pressure algometer and Von Frey Filaments) stimuli. Tests were performed over the skin at the most painful area on the lower limb and on the contralateral mirror‐image area. Prior to the tests, each subject was exposed to a training session which consisted of thermal (hot and cold), mechanical and pain thresholds. For a full description of the quantitative sensory testing see Data S1. For all thermal testing a 3 × 3 cm Peltier‐based computerized thermal stimulator was used (TSA, Medoc Ltd).

Notably, a manual muscle strength test using the American Spinal Injury Association (ASIA) motor score was also performed.

### 2.5 Assessment of inflammation

The degree of inflammation was assessed by measuring serum and disc levels of pro‐inflammatory cytokines. Serum levels of proinflammatory cytokines (IL‐1b, IL‐6, IL‐8, IL‐17, TNFα, INF‐g) were measured using the enzyme‐linked immunosorbent assay (ELISA) (Sigma^®^) (Matalka et al., 2005). Since cytokine levels (proteins) in the dissected disc tissue are extremely low (Shamji et al., 2010), we used the Real‐Time Quantitative Reverse Transcription Polymerase Chain Reaction (qRT‐PCR) method to detect cytokine RNA levels in the tissue (Heid et al., 1996). A full description of the procedure is provided in Data S2.

The relative expression of cytokines was calculated using the comparative threshold (Ct) method, as previously described (Livak & Schmittgen, 2001). Ct levels are inversely proportional to the amount of target nucleic acid in the sample (i.e. the lower the Ct level, the greater the amount of target nucleic acid in the disc sample). The Ct for each specific cytokine gene (target) was normalized using the delta Ct (ΔCt) method. The ΔCt is the distance between the specific cytokine target gene Ct and the housekeeping (GAPDH) gene Ct and is calculated as follows: ΔCt = Ct_target_ – Ct_GAPDH_.

### 2.6 Assessment of mechanical compression

MRI scans of enrolled patients, which were performed regardless of study participation, were evaluated by a single radiologist who was blinded to the subjects’ study data. The radiologist's evaluation consisted of determining the level of disc herniation, the specific nerve root compression, the type of disc herniation (protruded or extruded), and whether there was cephalic or caudal migration. The evaluation also included an estimation of the degree of nerve root compression and the size and location of herniation using the Michigan State University (MSU) classification, as well as a three‐dimensional volumetric analysis of the herniated disc.

#### 2.6.1 MSU classification

**FIGURE 1:**
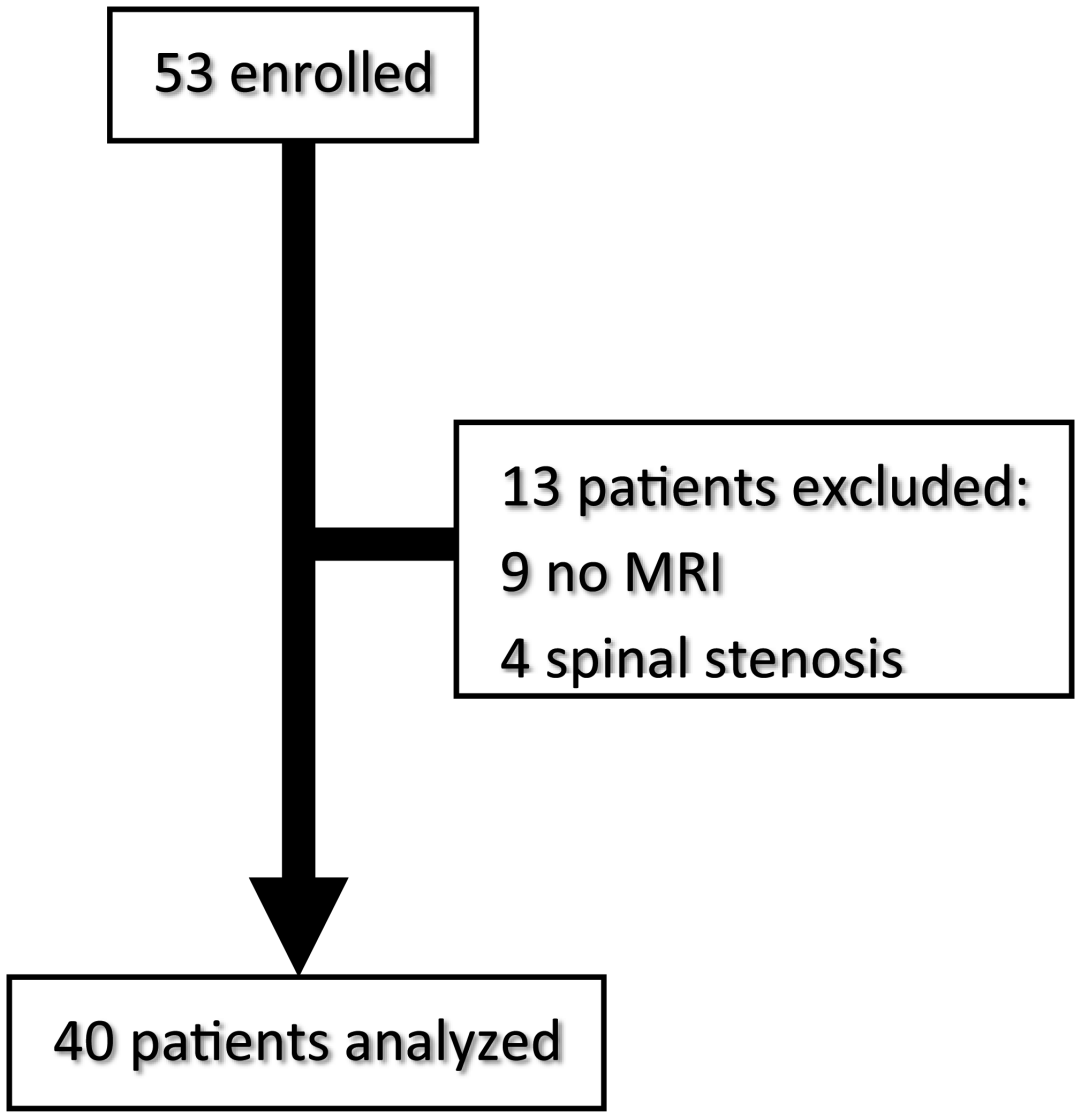
Flow Chart. Complete data were available from 40 (75%). Thirteen patients were excluded from the statistical analysis due to lack of MRI scans (9 patients) or additional pathology such as spinal stenosis (4 patients)

The MSU Classification takes into account both the size of disc herniation and its location within the spinal column (Figure 1). The measurements were taken from the T2 axial MRI images that matched best with the level of maximal herniation. For measuring the size of the herniation a single intra‐facet line was used as a reference point. In reference to the intra‐facet line, a determination was made as to whether the disc herniation extends up to, or less than, 50% of the distance from the non‐herniated posterior aspect of the disc to the intra‐facet line (size‐1), or more than 50% of that distance (size‐2). If the herniation extended altogether beyond the intra‐facet line, it was termed a size‐3 herniated disc. For the location assessment of the disc herniation, three reference points were placed along the intra‐facet line, dividing it into four equal quarters. The right and left central quadrants represented zone‐A and the right and left lateral quadrants represented zone‐B. A third zone‐C was represented at the level of the foramen by the area that extends beyond the medial margin of either facet joint. The determination, as into which zone the herniated nucleus intruded furthest, qualified the lesion as A, AB, B or C zoned (Mysliwiec et al., 2010).

#### 2.6.2 Three‐dimensional volumetric analysis

Herniated disc areas were measured on each sagittal section located between the lateral margins of each pedicle. Reference lines were drawn between the endpoints of the posterior edges of the superior and inferior endplates on each sagittal section. Measurements of the area of the herniated discs were obtained from each MRI sagittal section using the ‘region of interest‐polygon’ function of our picture archiving and communication system (PACS). Volume (mm^3^) was calculated by measuring the area (mm^2^) of each sagittal image and multiplying this value by the slice thickness (mm) (Seo et al., 2016).

### 2.7 Microbiology testing

The total DNA was extracted from the biopsy samples. DNA extraction was performed using the QIAamp DNA Mini Kit (Qiagen) according to the manufacturer's instructions. Two sets of primers were used in order to amplify different broad‐wide bacterial regions in the gene encoding for the 16S ribosomal RNA in the extracted DNA (Harmsen et al., 2003; Rothman et al., 2002). PCR products were separated by electrophoresis in ethidium bromide stained 2% agarose gels, and were then sequenced on a 3130 Genetic Analyzer capillary electrophoresis DNA sequencer (Applied Biosystems) and analysed using the basic local alignment search tool (BLAST). Additionally, biopsies were homogenized and plated for aerobic and anaerobic cultures according to clinical microbiology procedures handbook guidelines (Leber, 2016).

### 2.8 Assessment of pain processing

Pain modulation was quantified by the dynamic psychophysical paradigms of temporal summation (TS) and conditioned pain modulation (CPM). The tests were performed on the hands. For TS, noxious heat stimuli were given to the most painful area on the subjects’ lower limb using the 3 × 3 cm Peltier‐based computerized thermal stimulator (TSA, Medoc Ltd). The baseline temperature was 32°C and increased at a rate of 2°C/sec up to a destination temperature of 46.5°C and lasted for 2 min. Subjects, unaware of the temperature in each type of stimulus, were instructed to continually rate the pain intensity using a computerized visual analog scale (Co‐VAS). Individual temporal summation of heat pain was calculated as a subtraction of the lowest pain rating (i.e. the nadir affect which occurs after approximately 60 s) from the last pain rating (after 2 min). Hence, a positive value indicated a temporal summation process, and a negative value indicated an adaptation (Suzan et al., 2015).

Conditioned pain modulation comprised of applying first a heat test stimulus (47°C) to the dominant hand for 4 s, which was confirmed as painful by all patients. This was followed by simultaneous administration of a conditioning stimulus (immersing the non‐dominant hand in a 10°C cold water bath) and the same heat test stimuli. A consecutive heat test stimulus was given to the dominant hand after 30 s of non‐dominant hand immersion. Subjects were asked to verbally rate their perceived heat pain on a scale of 0–100 (0‐ no pain and 100‐ worst pain imaginable) at the end of each heat pain stimulus. The difference in pain intensity between the two heat test stimuli reflected the magnitude of CPM (Treister et al., 2013). Positive values indicate greater CPM efficiency.

### 2.9 Psychological assessment

The psychological assessment consisted of three questionnaires which were completed prior to surgery: (i) the Pain Sensitivity Questionnaire (PSQ) which consists of 18 daily life situations (15 painful and 3 non‐painful). Participants rate how painful each situation would be for them on a 0–10 numeric rating scale ranging (Ruscheweyh et al., 2009, 2012). (ii) the Pain Catastrophizing Scale (PCS) which comprises 13 statements evaluating three dimensions of pain catastrophizing: rumination, magnification and helplessness. Participants are instructed to rate their agreement with each statement on a scale as 0 = ‘never’, 1 = ‘almost never’, 2 = ‘occasionally’, 3 = ‘almost often’, 4 = ‘often’ (Granot & Ferber, 2005; Sullivan et al., 1995); and (iii) Spielberger's State‐Trait Anxiety Inventory (STAI) which includes two sections of 20 sentences, evaluating state and trait anxiety (Spielberger & Barratt, 1972; Teichman & Malineck, 1978).

### 2.10 Statistical analysis

Descriptive statistics, presented as mean ± standard deviation and median with interquartile range (IQR: 25%–75%), were used for the demographic variables, pain intensities, pain duration, psychological questionnaires, cytokine levels, QST variables and imaging variables, as appropriate. Non‐continuous variables are presented as percentages. The distribution of each variable was examined with the Shapiro–Wilk normality test. Based on this, log transformations to improve normality were used in the pain intensity variables (minimal, average and maximal pain), questionnaires (SF‐MPQ, PCS, PSQ), physical examination (SLR), volume of herniated disc and cytokines levels in serum and disc tissue. Paired sample *t*‐tests were performed to compare QST and SLR between the painful and non‐painful legs. Pearson correlations were performed to assess relationships between all cytokines in the serum, all cytokines in the disc samples and the relationships between cytokines in the serum and disc tissue together. In addition, multiple Pearson or Spearman (for ordinal scale variables) correlations were performed to assess the relationships between clinical pain variables (minimal, average and maximal pain intensities and SF‐MPQ), serum and disc tissue cytokine levels, imaging parameters (herniation volume, herniation location and neural compression), psychophysical parameters (CPM and TS) and psychological factors (PSQ, PCS and STAI). Benjamini‐Hochberg corrections were used in multiple correlations. Parameters were selected as candidates for the multivariate analysis on the basis of: (i) Level of significance from the univariate analysis; and (ii) Parameters that reduce multi co‐linearity. Models were developed using stepwise regressions to estimate the association between all pain variables and QST variables, pain‐related psychological questionnaires, types of pain, physical examination, imaging variables and cytokine levels. Statistical significance was determined as a *p* value of <0.05. SPSS version 25 was used for all statistical analysis. For a stepwise regression of 20 variables, with a medium effect size (f^2^ = 0.15), *α *≤ 0.05 and power of 0.80, the required sample size was 157 patients (G*Power statistical analysis; Faul et al., 2007).

### 2.11 Data availability

Additional research data which are not included in this article due to space limitations will be shared by request from any qualified investigator.

## 3 RESULTS

### 3.1 Patients

Complete data were available from 40 (75%) patients (15 female). Thirteen patients were excluded from the statistical analysis due to lack of MRI scans (9 patients) or additional pathology such as spinal stenosis (4 patients) (Figure 1). The study participants’ mean age was 44.8 ± 16.3 years and median (IQR) weight was 78.5 (73–90) kg. Disc herniations were most prevalent at L4‐5 and, L5‐S1 levels (42.5% each), followed by L3‐4 (10%), L2‐3 and L1‐2 (2.5%, each). They were equally distributed between the left and the right sides. Pain duration was 34 ± 22.6 weeks. Mean pain intensity was 57.1 ± 23.5 (0–100 NPS). At the time of testing 32 (80%) patients reported moderate to severe pain intensity (NPS > 4). All included patients did not undergo previous back surgery. Table 1 summarizes the minimal, mean and maximal pain intensities and the SF‐MPQ scores.

**TABLE 1:** Pain assessment (NRS and SF‐MPQ) and scores on pain related questionnaires

Pain measure	Mean ± SD	Median (IQR)
Pain durations (weeks)	34 ± 22.6	27(14.5–52)
Minimal pain (0–100 NPS)	26.6 ± 25.4	20 (5.25–42.5)
Average pain (0–100 NPS)	57.1 ± 23.5	60 (40–75)
Maximal pain (0–100 NPS)	76 ± 23.5	80 (67.5–90)
SF‐MPQ (0–45)	22.7 ± 9.06	23.5 (18–29.5)
SF‐MPQ sensory (0–30)	16.3 ± 6.65	17 (12.8–21)
SF‐MPQ affective (0–15)	6.38 ± 3.12	7 (4–8.25)
PSQ Score (0–10)	4.18 ± 1.77	3.79 (2.86–5.23)
PCS total Score (0–52)	34.8 ± 10.4	37 (28.8–43.3)
PCS – Rumination Score (0–16)	11.2 ± 3.94	12 (8–14)
PCS – Magnification Score (0–12)	6.88 ± 2.89	7 (5.75–9)
PCS – Helplessness Score (0–24)	16.7 ± 5.04	18 (13.8–20)
STAI total score (40–160)	79.7 ± 19.9	77 (68.3–92.5)
STAI – state anxiety score (20–80)	42.5 ± 11.5	41.5 (38.5–50.3)
STAI – trait anxiety score (20–80)	37.2 ± 10.8	35.5 (28.8–45)

Abbreviations: PCS, Pain Catastrophizing Questionnaire; PSQ, Pain Sensitivity Questionnaire; SF‐MPQ, Short‐Form McGill Pain Questionnaire; STAI, Spielberger's State‐Trait Anxiety Inventory.

### 3.2 Diagnosis of neuropathic pain

All 40 subjects were diagnosed with definite neuropathic pain according to the IASP definition (26). All patients had pain in a distribution of a defined dermatome, they all had positive SLR tests and at least one additional abnormal sensory test in the bedside sensory examination or the QST. QST results were considered abnormal if deviated from the DFNS norms (i.e. 2 SD away from the average) (Magerl et al., 2010). Additionally, MRI findings confirmed the diagnosis (Data S3 presents the results of all tests). Notably, reduction of muscle strength was noticeable in 22 out of the 40 subjects, although further supports the presence of neurological (radicular) abnormality, muscle weakness is not criteria requirement for diagnosing neuropathic pain according the IASP definition (Scholz et al., 2019).

### 3.3 Assessment of inflammation

**FIGURE 2:**
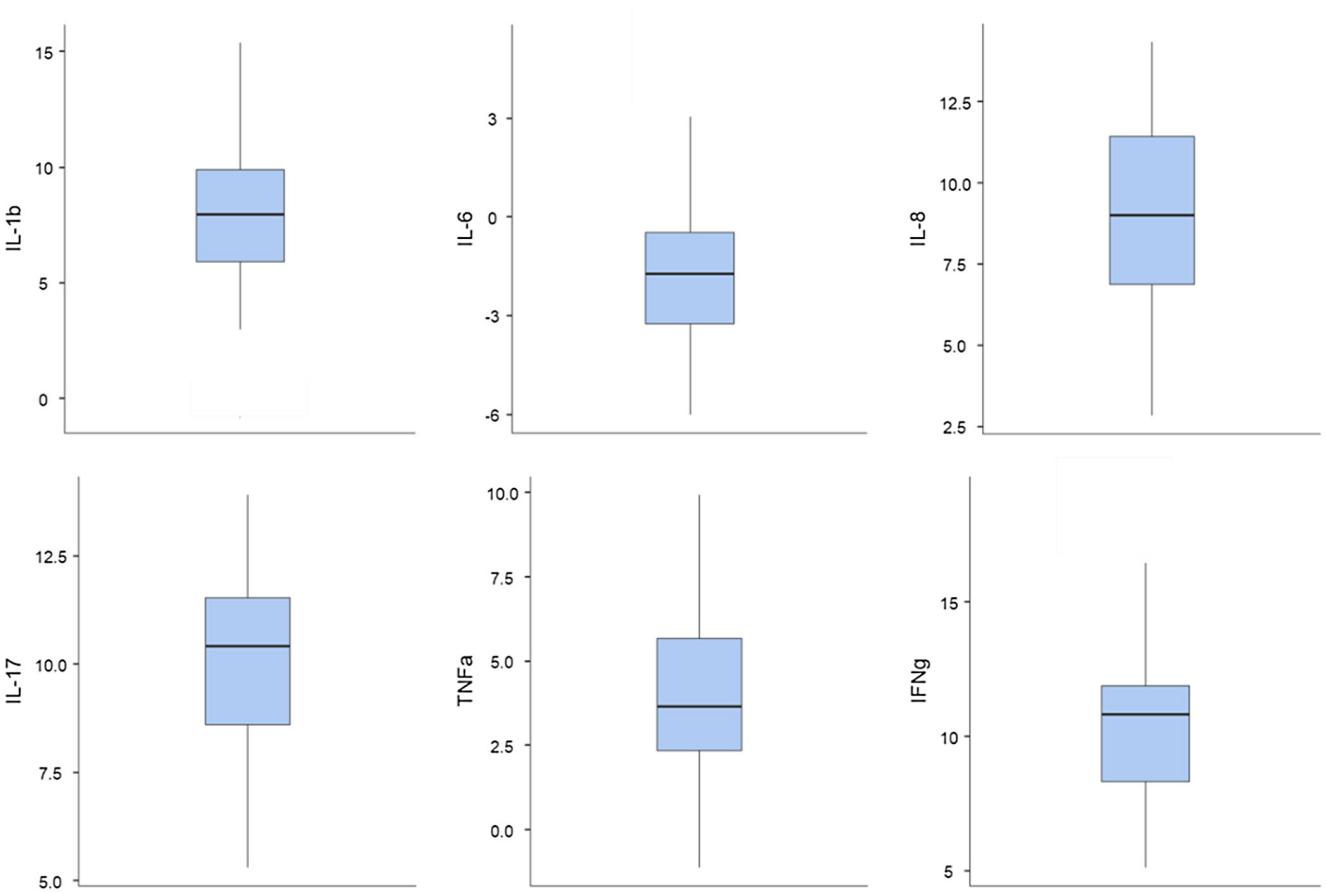
Disc tissue cytokines ΔCt. The ΔCt of each cytokine gene is shown in relation to the housekeeping gene (GAPDH). The ΔCt is the distance between the specific cytokine target gene Ct and the housekeeping gene Ct and is calculated as follows: ΔCt = Ct_target_ – Ct_GAPDH_. Note that higher negative values illustrate higher concentrations of the target cytokine gene

All pro‐inflammatory cytokines were expressed in serum and disc samples of all study subjects. The highest cytokine concentration (pg/ml; median [IQR] in the serum was measured for IL‐1b (15.3 [11–40.7]), followed by IL‐8 (15.2 [10.6–19.2]), IL‐6(8.29 [6.4–61.7]), TNFα (6.07 [5.34–7.68]), IL‐17 (4.51 [3.73–5.18] and IFNg (3.14 [2.75–4.13]). Figure 2 shows the ΔCt of each cytokine obtained from the disc tissue in relation to the GAPDH gene. Importantly, significant positive internal correlations were found between almost all serum cytokine levels (except IFNg) and most disc tissue cytokine levels. Several, although not all, cytokines in serum and disk samples also correlated with each other (Data S4–S6). Nonetheless, none of the serum or disc tissue cytokine levels correlated with any of the pain measures (pain intensities after log transformations and SF‐MPQ scores) and pain duration.

### 3.4 Assessment of mechanical compression

Levels and sides of disc herniations are presented in Data S3. Ninety percent of the discs were extruded and 55% migrated cranially or caudally. Most herniations (75%) were located in the central canal zone, followed by foraminal (12.5%), subarticular (10%) and extra‐foraminal (2.5%) zones. The mean ± SD volume of the herniations per the three‐dimensional analysis was 939 ± 712 mm^3^ ranging from 201 to 3472 mm^3^. The most frequent (42.5%) zone of the MSU classification was 2‐AB (central and subarticular zones with moderate nerve root compression). Table 2 summarizes the imaging parameters and the MSU classification distributions. Disc herniation volume positively correlated to the degree of spinal canal narrowing (*r* = 0.352, *p* = 0.028) and the anterior‐posterior component of the MSU classification (*r* = 0.614, *p *< 0.001), indicating that larger herniation volume is associated with grater posterior transition and higher spinal canal narrowing. Additionally, the anterior‐posterior and medial‐lateral components of the MSU classification negatively correlated with each other (*r* = −0.477, *p* = 0.002).

**TABLE 2:** Subjects’ MSU classification distribution

MSU classification	*N* (%)
1‐A	0 (0%)
1‐B	3 (7.5%)
1‐C	2 (5%)
2‐A	2 (5%)
2‐B	4 (10%)
2‐C	3 (7.5%)
2‐AB	17 (42.5%)
3‐A	4 (10%)
3‐B	0 (0%)
3‐AB	5 (12.5%)
Total	40 (100%)

No correlations were found between any of the MRI factors (herniation level, herniation type, migration, location and volume) and all pain intensities (NRS after log transformations and SF‐MPQ).

### 3.5 Assessment of bacterial infection

All disc samples were evaluated for bacterial infection. All aerobic and anaerobic cultures were negative for bacterial infection. In addition, from all of the 40 herniated disc samples only one was positive in the PCR essay for P. Acnes.

### 3.6 Assessment of pain processing

**FIGURE 3:**
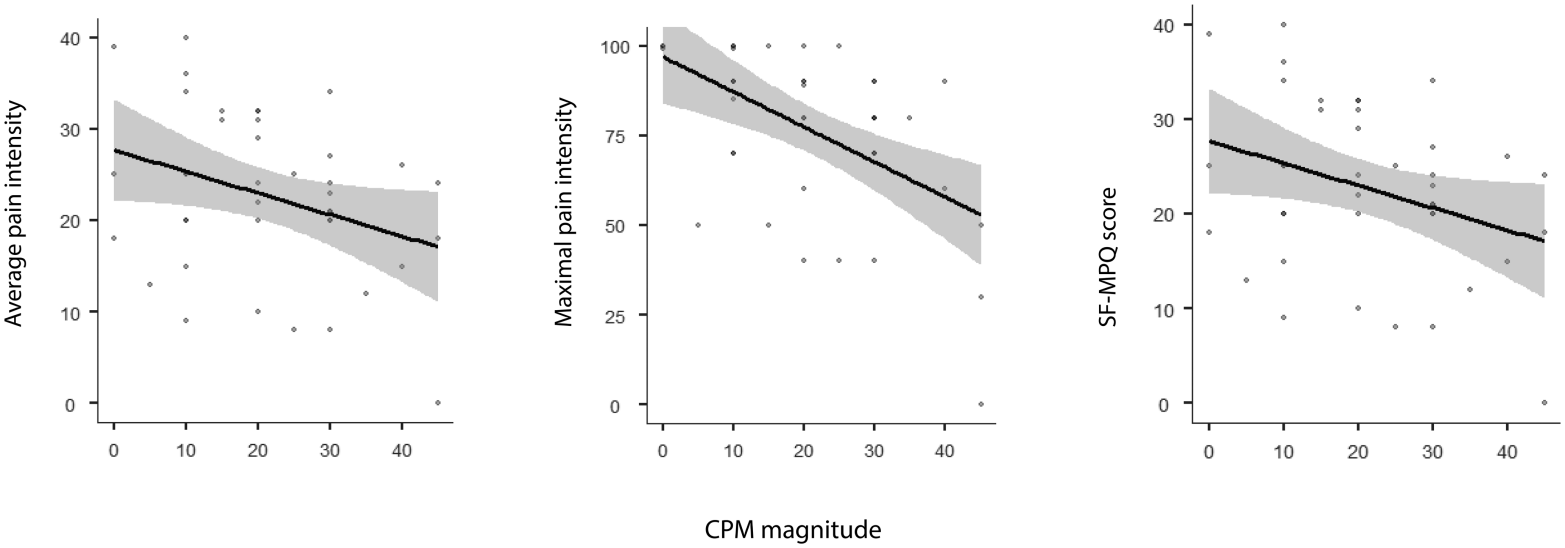
CPM correlation with pain variables. CPM magnitude negatively correlated with average pain ratings, maximal pain ratings and with the SF‐MPQ score

Magnitudes of CPM and TS (mean ± SD) were 21.4 ± 12.6 and 28.2 ± 18.5 respectively. CPM magnitude negatively correlated with average (*r* = −0.473, *p* = 0.002) and maximal (*r* = −0.455, *p* = 0.005) pain ratings and with the SF‐MPQ score (*r* = −0.421, *p* = 0.007) (Figure 3). No correlations were found between TS and any of the clinical pain measurements, and between TS and CPM with pain duration.

### 3.7 Psychological assessment

Scores of the psychological questionnaires (PSQ, PCS and STAI) are summarized in Table 1. They indicate that the patients had moderate self‐reported pain sensitivity, trait anxiety slightly below and state anxiety slightly above the clinical anxiety cut‐off point of 39–40 points (Knight et al., 1983), and significant pain catastrophizing with 75% of subjects scoring above 30 points on the total PCS (corresponding to the 75th percentile of PCS distribution scores in clinic samples of chronic pain patients; Sullivan et al., 1995).

**FIGURE 4:**
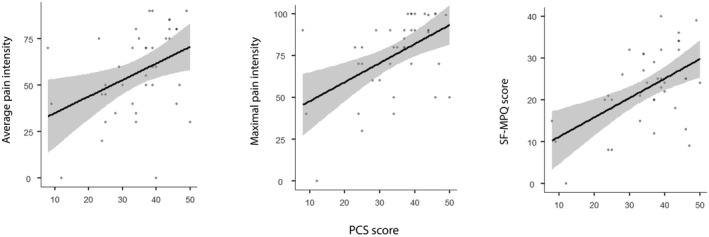
PCS correlation with pain variables. PCS moderate positively correlated with average pain ratings, maximal pain ratings and with the SF‐MPQ score

Positive moderate correlations were found between the PCS scores and pain measures (Table 3 and Figure 4). No correlations were found between PSQ and STAI scores and clinical pain variables. Additionally, correlations were found between all psychological questionnaires and pain duration.

**TABLE 3:** Correlation between clinical pain ratings and scores on psychological questionnaires

	PCS				PSQ	STAI		
	Rumination	Magnification	Helplessness	Total	Total	State	trait	total
Minimal pain	0.137	0.093	0.045	0.087	0.118	−0.011	−0.150	−0.090
Average pain	0.300	0.267	0.460**	0.429**	0.223	0.066	0.112	0.104
Maximal pain	0.220	0.224	0.561***	0.453**	0.055	0.078	0.067	0.064
SF‐MPQ sensory	0.328*	0.288	0.383*	0.459**	0.159	0.196	0.157	0.228
SF‐MPQ affective	0.555***	0.348*	0.506***	0.551***	0.075	0.173	0.271	0.301
SF‐MPQ total	0.450**	0.358*	0.417**	0.447**	0.128	0.194	0.201	0.257

Note

Spearman correlations, *p < 0.05, **p < 0.01, ***p < 0.001.

### 3.8 Regression models

A stepwise regression model was used for estimating the degree to which average pain, maximal pain and MPQ scores may be explained by variance in age, gender, PCS and CPM magnitude. The following regression equations were used: Average pain intensity = 43.24 + (−0.82 × CPM) + (5.877 × gender [female = 1, male = 0]). The model was found to be significant for CPM (*p* = 0.001) and gender (*p* = 0.029) with an adjusted R^2^ of 0.443. Thus, low CPM magnitude and female gender are associated with greater average pain intensity. Maximal pain intensity = 63.17 + (−0.73 × CPM) + (0.82 × PCS). The model was significant for CPM (*p* = 0.007) and PCS (*p* = 0.012) with an adjusted R^2^ of 0.38. Thus, low CPM magnitude and greater PCS scores are associated with greater maximal pain intensity.

MPQ = 6.6 + (0.46 × PCS). This model was significant for PCS (*p* = 0.000) with an adjusted R^2^ of 0.28, meaning that greater PCS scores are associated with greater MPQ scores.

## 4 DISCUSSION

The present exploratory study attempted to evaluate the relationships between the intensity of pain and factors potentially contributing to the pain experience in patients with lumbar radiculopathy. Unlike many other studies which typically tested a single factor in dispersed populations of patients, in the present study five factor categories were assessed in one well defined group of patients with confirmed painful radiculopathy. Understanding the underlying pathophysiology may lead to better clinical decision making with regards to the use of anti‐inflammatory drugs, psychological interventions, surgery or other analgesic modalities in patients with painful lumbar radiculopathy mo. Two main findings emerge from the study. First, pain intensity was not associated with pro‐inflammatory serum and disc cytokine levels nor with MRI disc‐size measures. Second, rather than these objective parameters, pain intensity correlated with the patients’ reported measures of pain catastrophizing and with their CPM magnitude.

### 4.1 The role of inflammatory in painful radiculopathy

The role of inflammation in sciatica has been reported in numerous studies (Molinos et al., 2015). Intervertebral disc cells can produce proinflammatory mediators which in turn results in the recruitment of macrophages, lymphocytes and the secretion of mediators such as IL‐1b, IL‐8, IL‐6, IL‐17, TNFα, IFNg, PGE2 along with nerve growth factor and substance P production (Molinos et al., 2015). A systematic review of 16 studies (1212 patients) concluded that there is insufficient evidence to draw firm conclusions regarding the relationship between inflammation and clinical symptoms (Jungen et al., 2019). Of all measured cytokines, only IL‐21 obtained from disc tissue and TNFα from both serum and disc tissue demonstrated strong and medium correlations, respectively, with pain severity (VAS>4). Positive correlations were also demonstrated between the same two cytokines (Chen et al., 2017) as well as with serum IL‐8 (Pedersen et al., 2015) in follow‐up studies. Moreover, high serum IL‐6 levels were associated with less favorable recovery in patients with lumbar radicular pain (Schistad et al., 2014). Contrasting findings were reported by Andrade et al., who reported no differences in cytokine levels between painful and non‐painful patients, before and 1 year after back surgery (Andrade et al., 2013). They concluded that ‘cytokines may not play a leading role in maintaining a painful generating network’ (Andrade et al., 2013). This is further supported by two systematic reviews which showed only inconsistent, small and short‐lived analgesic effects of NSAIDs (Rasmussen‐Barr et al., 2016) and epidural corticosteroid injections (Pinto et al., 2012) in patients with sciatica.

Our study, demonstrated significant levels of proinflammatory cytokines in serum samples and proinflammatory cytokine genes expression in disc tissues, similar to those found in other studies on patients with sciatica (Pedersen et al., 2015; Wang et al., 2016; Zu et al., 2016). Nonetheless, contrary to our hypothesis, cytokine levels did not correlate with levels of pain, suggesting that inflammation does no play a major role in determining the intensity of radicular pain. Moreover, the fact that no correlations were found between disk and serum proinflammatory cytokine levels and pain duration suggests that this is likely true for both acute and chronic radicular pain.

### 4.2 The role of mechanical compression in painful radiculopathy

Controversy regarding the contribution of nerve root compression to pain in lumbar radiculopathy also exists. Animal studies have linked nerve root compression with nociceptive behavior (mechanical allodynia and thermal hyperalgesia) and sustained dorsal root ganglion (DRG) neural discharges (Howe et al., 1977), and progressive nerve root ligation strain and amplified mechanical allodynia (Winkelstein & DeLeo, 2004b). However, other studies have found opposing findings showing that compression of a normal nerve root causes only a brief nerve root discharge and is too short to cause radicular pain (Howe et al., 1977; Mlekusch et al., 2016).

Human studies share a similar dispute to the contradictory findings from animal models. Extraforaminal disc herniations that directly compress the DRG have been reported to cause more leg pain and walking limitations than disc herniations with no DRG compression (Ohmori et al., 2001). Moreover, patients with radiological findings confined to nerve root displacement are more likely respond to local steroidal injections than patients with higher grades of compression (Ghahreman & Bogduk, 2011). In contrast, other studies have failed to show such anatomical correlations (Splettstößer et al., 2017). For instance, radiological findings in follow‐up imaging studies did not correlate with clinical symptoms (El Barzouhi et al., 2013), while positive imaging spine pathologies increase with age (El Barzouhi et al., 2013) even in pain‐free subjects (Baker, 2014; Boos et al., 2000;). Congruently, no correlations between accepted measures of disc herniation volume and location/size and pain intensity (and degree of muscle weakness) were found in our study. ‘Less painful’ foraminal (Splettstößer et al., 2017) or ‘more painful’ extraforaminal (Ohmori et al., 2001) disc locations cannot explain this finding because each location accounted for only a minority of the patients (15% and 10% respectively). Alternatively, since MRIs were performed in the supine position, they may fail to identify changes in the size of the central lumbar canal and the neural foramen caused by axial load in an upright position (Nowicki et al., 1990), and consequently may not be sensitive enough to accurately measure nerve root compression. Finally, we chose to use standardized disc measures rather than a radiologist's assessment of nerve root compression due the marked variability and high prevalence of interpretive errors in radiologists’ reports of MRI examinations of the lumbar spine. According to Herzog et al. (Herzog et al., 2017), the largest interpretive miss rate among eight common spine pathologies, was nerve root involvement.

### 4.3 Assessment of pain processing

CPM is widely used in experimental studies and is thought to assess pain modulation capacity (Yarnitsky, 2010). Low CPM efficiency was shown to have a predictive value for acute and chronic post‐operative pain (Yarnitsky, 2010) and is impaired in patients suffering from chronic pain according to a systematic review (Lewis et al., 2012). CPM has been studied in the context of low back pain, not specifically painful radiculopathy, and yielded mixed results. The findings from our study, not only show correlations between pain and reduced CPM magnitude, but also demonstrate a predictive capacity of average pain by CPM, further exemplifying the role of pain modulation in painful radiculopathy.

In contrast, TS, a phenomenon seemingly related to central sensitization and chronic pain, did not correlate with pain intensity in our patients. Enhanced TS has been reported in patients with back pain, fibromyalgia (Staud et al., 2001), temporomandibular pain (Maixner et al., 1998), and postoperative chronic pain (Petersen et al., 2015). We could find only two previous reports on the assessment of TS in patients with radicular pain but neither of them looked for associations between pain intensity and TS magnitude (Suzan et al., 2016).

### 4.4 Psychological assessment

Although psychological factors are strongly associated with back pain, out of the three questionnaires completed by our patients, only pain catastrophization showed significant associations with pain measures. The regression model in the present study showed that 15.4% of average pain and 25.5% of maximal pain ratings were attributed to pain catastrophizing, falling within the previously reported range of 7–31% ascription of catastrophization to chronic pain.

Notably, previous studies reported associations between pain catastrophizing and both CPM (Weissman‐Fogel et al., 2008) and TS (George et al., 2007). A positive association were also found in our patients between pain catastrophizing and CPM but not with TS, suggesting that pain catastrophizing can be associated with reduced endogenous pain inhibition. Associations between pain catastrophizing and the inflammatory system have also been reported, pointing to potential neuro‐immunologic mechanisms underlying pain. Similarly, we found in the current study positive correlations between the helplessness subscale of catastrophizing and serum levels of IL‐1b, IL‐6, TNFα and IFNg.

### 4.5 Study limitations

Several limitations should be noted: First the fact that no control group was added limited the statistics to within group analyses only. However, obtaining disc biopsies from patients undergoing back surgeries for other reasons was ethically challenging. Second: due to the small sample size the study should be considered as an exploratory study. Third: the fact that we have somewhat modified the TS component of the DFNS protocol, should be noted.

## 5 CONCLUSIONS

Study results suggest that pain catastrophizing, CPM and gender rather than objective measures of inflammation and imaging indicators of mechanical nerve compression seem to contribute to pain intensity in patients with painful radiculopathy. Larger scale studies are warranted for confirming these findings.

## CONFLICTS OF INTEREST

G. Samuelly‐Leichtag (PhD) reports no disclosures. E. Eisenberg (MD) reports no disclosures. Y. Zohar (MD, PhD) reports no disclosures. M. Andraous (MD) reports no disclosures. A. Eran (MD) reports no disclosures. G. Sviri (MD) reports no disclosures. O. Keynan (MD) reports no disclosures.

## Supporting information

Supplementary Material
