# Supplementary material for: Mechanism underlying painful radiculopathy in patients with lumbar disc herniation
Source: Eur J Pain. 2022 Apr 12;26(6):1269–81. doi: 10.1002/ejp.1947 (PMC10083974; doi:10.1002/ejp.1947)
Supplement: Supplementary file 1 — Supplementary Material [file EJP-26-1269-s001.docx]

**Supplemental data 1**

**Thermal detection thresholds**

Cold and warm detection thresholds were measured first (CDT, WDT) using the TSA II (MEDOC, Israel). A 3x3 cm2 thermode was placed over the most painful site over the subjects' leg and the mirror area of the contralateral leg. To determine cold/heat detection thresholds a set of six stimuli (three cold and three hot) were given with a baseline temperature of 32°C and a ramped stimuli of 1°C/s. Subjects were asked to press a button when noticing the first change in temperature (high or low). The mean threshold temperature of three consecutive measurements were calculated as the thermal detection thresholds.

**Thermal pain thresholds**

Similar the thermal detection thresholds, a 3x3 cm2 thermode was placed over the most painful site over the subjects' leg and the mirror contralateral leg. To determine cold pain (CPT) and heat pain thresholds (HPT) a set of six stimuli (three cold and three hot) were given with a baseline temperature of 32°C, a ramped stimuli of 1°C/s and 15 second rest time between stimuli. Subjects were asked to press a button when each of the stimulus (hot or cold) was perceived as minimally painful (equal to 1 on a 0-10 numeric pain scale). The stimuli were terminated as soon as the subject pressed the button and returned to baseline temperature with cut-off temperatures of 0°C and 50°C. The mean threshold temperature of three consecutive measurements were calculated as cold pain and heat pain thresholds.

**Mechanical detection and pain threshold**

The mechanical detection threshold (MDT) was measured with a standardized set of von Frey hairs (Optihair2-Set, Marstock Nervtest, Germany) that exert forces, upon bending, between 0.25 and 512 mN (1–2s contact time). Using the ‘‘method of limits’’, three threshold determinations were made over the skin on the most painful leg area and the contralateral leg, each with a series of ascending and descending stimulus intensities. The final threshold was calculated as the geometric mean of these three series.

The mechanical pain threshold (MPT) was measured using a standardized set of von Frey hairs. The stimulators were applied at a rate of 2 seconds on and 2 seconds off in an ascending order until the first percept of sharpness was reached. The final threshold was determined by the geometric mean of three series of ascending and descending stimuli.

**Vibration detection threshold**

The vibration detection threshold (VDT) test was performed with a Rydel–Seiffer grade tuning fork (64 Hz, 8/8 scale) that was placed over a bony prominence (lateral / medial malleolus or lateral / medial knee condyles, depending on the affected spinal dermatome) of both legs and left there until the subject could not feel the vibration any more. Vibration detection threshold was determined as a disappearance threshold with three stimulus repetitions.

**Pressure pain threshold**

Pressure pain threshold (PPT) test was performed over a muscle in the most painful area on the subjects' lower limb and the contralateral side using a pressure gauge device (FDN200, Wagner Instruments, USA) with a probe area of 1 cm2 that exerts forces up to 20 kg/cm2. The pressure pain threshold was determined with three series of ascending stimulus intensities, each applied as a slowly increasing ramp of 0.5 kg/cm2 s. Subjects were asked to verbally announce by saying the word "now" when the pressure stimulus was perceived as minimally painful (equal to 1 on a 0-10 numeric pain scale). The mean of the three pressure gauge device readings were determine as the PPT.

**Pressure pain rating**

Pressure pain sensitivity (PPR) was tested using the pressure gauge device (FDN200, Wagner Instruments, USA) with a probe area of 1 cm2 that exerts forces up to 20 kg/cm2. Three pressure noxious stimuli were given over the most painful area on the subjects' lower limb and the contralateral side for 2 seconds. The force of the noxious pressure stimulus was determined as 1 kg/cm2 above the mean PPT. Subjects were asked to verbally rate each stimulus on a 0-100 pain scale (0- no pain and 100- worst pain imaginable). Pressure pain sensitivity was calculated as the mean of all three pain ratings.

**Temporal summation**

Noxious heat stimuli were given to the most painful area on the subjects' lower limb using the 3 X 3 cm Peltier-based computerized thermal stimulator (TSA, Medoc Ltd, Ramat Yishai, Israel). The baseline temperature was 32ºC and increased at a rate of 2ºC/sec up to a destination temperature of 46.5ºC and lasted for 2 minutes. Subjects, unaware of the temperature in each type of stimulus, were instructed to continually rate the pain intensity using a computerized visual analog scale (Co-VAS). Individual temporal summation of heat pain was calculated as a subtraction of the lowest pain rating (i.e. the nadir affect which occurs after approximately 60 seconds) from the last pain rating (after two minutes). Hence, a positive value indicated a temporal summation process, and a negative value indicated an adaptation ([Suzan et al., 2015](#_ENREF_67)).

**Conditioned pain modulation**

DNIC efficiency was measured using the *conditioned pain modulation* (CPM) paradigm, which compares perceived pain intensity of a noxious stimulus alone ('test stimulus') and while another noxious stimulus is applied to a remote body part ('conditioning stimulus'). The test stimuli consisted of a noxious heat stimuli given to the thenar area of the dominant hand using the 3 X 3 cm Peltier-based computerized thermal stimulator (TSA, Medoc Ltd, Ramat Yishai, Israel). The baseline temperature was 32ºC and increased at a rate of 2ºC/sec up to a destination temperature of 47ºC and lasted for 4 seconds. The conditioning stimuli consisted of immersing the non-dominant hand in a 10ºC cold water bath. Three consecutive test stimuli were given every 15 seconds. Right after the second test stimulus terminated the subject were asked to immerse the contralateral hand in the 10ºC cold water bath. During each stimulus subjects were asked to verbally rate their perceived pain on a scale of 0-100 (0- no pain and 100- worst pain imaginable). DNIC efficiency was calculated by subtracting the mean pain ratings of the test stimulus alone from the mean pain ratings of the test stimulus during hand immersion. Thus, negative values indicated greater CPM efficiency.

**Supplemental data 2**

**Blood and tissue obtainment**

Blood samples were collected at the operation room right before the beginning of the surgery. The samples were let clot for 15 minutes and then were centrifuged at 4000 RPM for 15 minutes at a temperature of 4°c. Serum samples were separated, aliquoted and frozen at -70°c for later processing. Herniated disc tissues, which were dissected as part of the pre-planned surgery were collected in a sterile tube and divided in to four proximally equal samples. Two samples were frozen at -70°c for RNA extraction, one sample was delivered to the microbiology laboratory for culture and the fourth sample was placed in formalin for tissue processing and sectioning.

### Total RNA extraction and DNA removal

Total RNA was extracted from disc tissues using TRI Reagent (Sigma ®, Rehovot, Israel) according to manufacturer's instructions. Total RNA was pelleted, dried and re-suspended in diethyl pyrocarbonate (DEPC) treated water. RNA concentration was determined using spectrophotometer at wavelength of 260nm. RNA was treated with TURBO DNA-free™ DNase (Thermo Fisher Scientific Baltics UAB, Vilnius, Lithuania) according to manufacturer's instructions. This treatment is designed to remove contaminating DNA from RNA preparations, and subsequently remove DNase and divalent cations from the sample.

### cDNA synthesis using random primer reverse transcriptase (RT)

### RNA was reversely transcribed into complementary DNA (cDNA), using reverse transcriptase. Single-stranded cDNA was synthesized form RNA using M-MuLV reverse transcriptase (GoScript™, Promega Corporation, Madison, USA). 2µg of total RNA were incubated for two hours in 42ºC in reaction mix that contained RT reaction buffer, 1mM dNTPs (each), 1nmole of random hexamers, 50units of RNase inhibitor, 2µg BSA and 25units of M-MuLV RT in DEPC treated water.

*qRT-PCR:* qRT-PCR was performed using the Rotor-Gene SYBR Green PCR kit (QIAGEN, Germantown, MD) with the primer pairs (described in appendix 2) on Light Cycler Systems (Roche). The assay was calibrated using primer for glyceraldehyde-3-phosphate dehydrogenase (GAPDH) as the internal control. The cycle consisted of a 1-minute denaturation at 94°C, a 30-second annealing at 57°C, and a 30-second extension at 72°C.

**Forwards and reverse primers**

| **Primer** | **Nucleotide sequence** |
| --- | --- |
| hIL-1b F | 5'ACAGATGAAGTGCTCCTTCCA3' |
| hIL-1b R | 5'GTCGGAGATTCGTAGCTGGAT3' |
| hIL-6 F | 5'GGATTCAATGAGGAGACTTGCCT3' |
| hIL-6 R | 5'GTTCTGGAGGTACTCTAGGTATACCTC3' |
| hIL-8 F | 5'GAAGAGAGCTCTGTCTGGACCC3' |
| hIL-8 R | 5'TGAATTCTCAGCCCTCTTCAAAAACT3' |
| hIL-17 F | 5'GGAATCTCCACCGCAATGAGG3' |
| hIL-17 R | 5'TTGATGCAGCCCAAGTGGCG3' |
| hTNF-α F | 5'GAGGGAAGAGTTCCCCAGGG3' |
| hTNF-α R | 5'CACTCGGGGTTCGAGAAGAT3' |
| hIFN-g F | 5'GAAAAGCTGACTAATTATTCGGTAACTGACTTG3' |
| hIFN-g R | 5'CAGTTCAGCCATCACTTGGATGAG3' |
| hGAPDH F | 5'GGATATTGTTGCCATCAATGACCCC3' |
| hGAPDH R | 5'TTGCCATGGGTGGAATCATATTGG3' |

### Cytokine ELISA:

### Cytokine concentrations in serum were measured using commercial ELISA kits, and according to manufacturer's instructions. Briefely, MaxiSorp™ flat-bottom 96 well plates (Nunc) were coated with capture antibody diluted in PBS and were kept in 4°C overnight, blocked with blocking solution (1% BSA in PBST) for 1h and serum was applied to the wells. Plates were kept in 4°C overnight, washed three times with PBST and biotin-conjugated detection antibodies, diluted in blocking solution, were added. Plates were incubated for 2h at room temperature, washed three times with PBST and streptavidin-HRP was added for 20min. Plates were then washed three times with PBST and TMB was added. After color has developed, the reaction was stopped with 10% H_2_SO_4_ solution and plates were read in 450nm wavelength with reference wavelength of 630nm. Recombinant proteins, supplied with the kit were used for generation of standard curves, and cytokine concentration was calculated according to the standard curve.

**Supplemental data 3 - Diagnosis of neuropathic pain**

|  |  | **Bedside testing** | | | | **QST** | | | | | | | | **MRI** | |
| --- | --- | --- | --- | --- | --- | --- | --- | --- | --- | --- | --- | --- | --- | --- | --- |
| **SN** | **MPD** | **LT** | **PP** | **AL** | **SLR** | **WDT** | **CDT** | **WPT** | **CPT** | **MDT** | **MPT** | **VDT** | **PPT** | **Nerve root compression** | **Herniation level (side)** |
| **1** | L5 | - | N | N | P 60 | - | N | N | N | - | N | - | + | L5 | L4-L5(L) |
| **2** | L5 | N | N | N | P 25 | N | N | N | N | - | N | - | + | L5 | L4-L5(L) |
| **3** | S1 | N | N | N | P 45 | N | N | N | N | N | N | - | + | S1 | L5-S1(R) |
| **4** | L5 | N | N | + | P 32 | - | N | - | - | N | N | - | N | L5 | L4-L5(L) |
| **5** | L5 | N | N | N | P 5 | N | N | N | N | N | N | N | N | L5 | L4-L5(L) |
| **6** | S1 | N | N | N | P 45 | + | N | N | N | N | N | N | N | S1 | L5-S1(R) |
| **7** | L5 | + | N | N | P 50 | + | N | N | N | N | N | - | N | L5 | L4-L5(L) |
| **8** | L5 | N | N | N | P 40 | + | N | + | N | N | N | - | N | L5 | L4-L5(R) |
| **9** | S1 | N | N | + | P 20 | N | N | + | N | - | N | - | + | S1 | L5-S1(R) |
| **10** | L3 | N | N | N | P 30 | + | N | N | - | N | N | - | N | L2+L3 | L2-L3(R) |
| **11** | S1 | - | + | N | P 35 | + | N | N | N | N | N | - | N | S1 | L5-S1(L) |
| **12** | S1 | N | N | N | P 40 | N | N | N | - | N | N | - | N | S1 | L4-L5(R) |
| **13** | S1 | N | N | N | P 10 | N | N | N | - | + | N | - | N | S1 | L5-S1(L) |
| **14** | L5 | - | N | N | P 35 | N | N | N | N | - | N | - | N | L5 | L4-L5(R) |
| **15** | L5 | N | N | N | P 35 | N | N | N | N | N | N | - | N | L5+S1 | L4-L5(R) |
| **16** | L3 | N | N | N | P 45 | + | N | N | N | - | N | N | N | L3 | L3-L4(L) |
| **17** | L3 | N | N | N | P 45 | N | N | N | N | - | N | - | N | L3 | L3-L4(L) |
| **18** | L5 | - | N | + | P 20 | N | N | N | N | - | N | N | N | L5 | L4-L5(L) |
| **19** | S1 | N | - | N | P 30 | + | N | N | N | N | N | N | N | S1 | L5-S1(L) |
| **20** | S1 | N | N | N | P 35 | N | N | N | N | - | N | N | N | S1 | L5-S1(R) |
| **21** | L4 | - | - | + | P 20 | N | N | N | - | - | N | N | N | L3+L4 | L3-L4(R) |
| **22** | L4 | - | - | N | P 40 | N | N | N | N | - | N | N | + | L4 | L3-L4(L) |
| **23** | L4 | - | - | N | P 30 | N | N | N | N | N | N | N | N | L4 | L4-L5(L) |
| **24** | L4 | N | N | N | P 30 | N | N | N | N | N | N | N | + | L4 | L4-L5(L) |
| **25** | L5 | - | - | N | P 45 | + | N | N | - | N | N | N | N | L5 | L4-L5(L) |
| **26** | L5 | N | N | N | P 25 | N | N | N | - | N | N | N | N | L5 | L4-L5(R) |
| **27** | S1 | - | - | N | P 30 | N | N | + | N | N | N | N | N | S1 | L5-S1(R) |
| **28** | S1 | - | - | N | P 30 | N | N | N | N | N | N | N | + | S1 | L5-S1(R) |
| **29** | S1 | - | N | N | P 20 | + | N | N | N | N | N | - | + | S1 | L5-S1(L) |
| **30** | S1 | N | N | N | P 25 | N | N | N | N | N | N | - | N | S1 | L5-S1(R) |
| **31** | S1 | N | N | N | P 15 | + | N | N | N | N | N | - | N | S1 | L5-S1(L) |
| **32** | L5 | - | - | N | P 40 | N | N | N | N | N | N | N | N | L5 | L4-L5(R) |
| **33** | L1 | - | - | N | P 45 | + | N | N | N | N | N | N | N | L1 | L1-L2(R) |
| **34** | L5 | - | - | N | P 20 | + | N | N | N | N | N | N | N | L5 | L4-L5(R) |
| **35** | S1 | - | - | N | P 30 | + | N | N | N | N | N | N | N | S1 | L5-S1(L) |
| **36** | S1 | - | - | N | P 70 | N | N | N | N | N | N | N | N | S1 | L5-S1(L) |
| **37** | S1 | - | - | N | P 35 | N | - | N | N | N | N | - | - | S1 | L5-S1(R) |
| **38** | S1 | N | N | N | P 32 | N | N | - | - | N | N | N | N | S1 | L5-S1(R) |
| **39** | S1 | - | - | N | P 20 | + | N | N | N | N | N | N | + | S1 | L5-S1(R) |
| **40** | L5 | N | N | N | P 10 | + | N | N | N | N | N | N | + | L5 | L4-L5(R) |

SN= subject number, MPD= most painful dermatome, LT= light touch, PP= pinprick, AL= allodynia, SLR= straight leg raise, WDT= warm detection threshold, CDT= cold detection threshold, WPT= warm pain threshold, CPS= cold pain threshold, MDT= mechanical detection threshold, MPT= mechanical pain threshold, VDT= vibration detection threshold, PPT= pressure pain threshold. . + = positive results, - = negative results, N= normal results (according to the DFNS norms)

**Supplemental data 4– Serum cytokines levels and correlations**

|  | pg/mL | IL-6 | IL-8 | IL-17 | TNFα | IFNg |
| --- | --- | --- | --- | --- | --- | --- |
| IL-1b | 15.3 (11-40.7) | 0.866*** | 0.692*** | 0.745*** | 0.859*** | 0.22 |
| IL-6 | 8.29 (6.4-61.7) |  | 0.581*** | 0.61*** | 0.753*** | 0.226 |
| IL-8 | 15.2 (10.6-19.2) |  |  | 0.71*** | 0.778*** | 0.058 |
| IL-17 | 4.51 (3.73-5.18) |  |  |  | 0.833*** | 0.495** |
| TNFα | 6.07 (5.34-7.68) |  |  |  |  | 0.286 |
| IFNg | 3.14 (2.75-4.13) |  |  |  |  |  |

Values are expressed as pg/mL, median (IQR). Pearson's tests were used to assess the correlation between cytokines. *P<0.05, **P<0.01, ***P<0.001.

**Supplemental data 5 – Disc tissue cytokine correlations**

|  | IL-6 | IL-8 | IL-17 | TNFα | IFNg |
| --- | --- | --- | --- | --- | --- |
| IL-1b | 0.23 | 0.729*** | 0.404** | 0.539*** | 0.092 |
| IL-6 |  | 0.171 | 0.491** | 0.592*** | 0.454** |
| IL-8 |  |  | 0.455** | 0.509*** | 0.323* |
| IL-17 |  |  |  | 0.573*** | 0.251 |
| TNFα |  |  |  |  | 0.446** |
| IFNg |  |  |  |  |  |

Pearson's tests were used to assess the correlation between cytokines. *P<0.05, **P<0.01, ***P<0.001.

**Supplemental data 6**

Serum IL-8 was correlated with disc samples of IL-1b (r=0.438, P=0.009), IL-17 (r=0.336, P-0.049), and TNFα (r=0.417, P=0.013), and serum IL-1b was correlated with disc sample IL-17 (r=0.406, P=0.015).

Positive correlations were also found between PCS sub domain of helplessness and serum levels of IL-1b(r=0.388, P=0.021), IL-6(r=0.351, P=0.038), TNFa (r=0.421, P=0.021) and IFNg (r=0.353, P=0.037).

Significant positive correlations were found between temporal summation and disc levels of IL-1b and IL-8 (r=0.452, P=0.003; r=0.435, P=0.005; respectively). No other correlations between QSTs and imaging parameters were found.
